# Towards Sustainable Packaging Using Microbial Cellulose and Sugarcane (*Saccharum officinarum* L.) Bagasse

**DOI:** 10.3390/ma17153732

**Published:** 2024-07-27

**Authors:** Cláudio José Galdino da Silva Junior, Alexandre D’Lamare Maia de Medeiros, Anantcha Karla Lafaiete de Holanda Cavalcanti, Julia Didier Pedrosa de Amorim, Italo José Batista Durval, Yasmim de Farias Cavalcanti, Attilio Converti, Andréa Fernanda de Santana Costa, Leonie Asfora Sarubbo

**Affiliations:** 1 Instituto Avançado de Tecnologia e Inovação (IATI), Rua Potyra, 31, Prado, Recife 50751-310, Brazil; claudio.junior@iati.org.br (C.J.G.d.S.J.); alexandre.medeiros@iati.org.br (A.D.M.d.M.); anantchalafaiete@gmail.com (A.K.L.d.H.C.); julia.amorim@iati.org.br (J.D.P.d.A.); italo.durval@iati.org.br (I.J.B.D.); yasmimfcavalcanti9@gmail.com (Y.d.F.C.); andrea.santana@ufpe.br (A.F.d.S.C.); 2 Rede Nordeste de Biotecnologia (RENORBIO), Universidade Federal Rural de Pernambuco, Rua Dom Manuel de Medeiros, s/n, Dois Irmãos, Recife 52171-900, Brazil; 3 Escola Icam Tech, Universidade Católica de Pernambuco (UNICAP), Rua do Príncipe, 526, Boa Vista, Recife 50050-900, Brazil; 4 Department of Civil, Chemical and Environmental Engineering, University of Genoa (UNIGE), Pole of Chemical Engineering, Via Opera Pia, 15, 16145 Genoa, Italy; 5 Centro de Comunicação e Design, Centro Acadêmico da Região Agreste, Universidade Federal de Pernambuco (UFPE), BR 104, km 59, s/n, Nova Caruaru, Caruaru 50670-901, Brazil

**Keywords:** bacterial cellulose, biomass, packaging design, recycling, waste reduction

## Abstract

The high consumption of packaging has led to a massive production of waste, especially in the form of nonbiodegradable polymers that are difficult to recycle. Microbial cellulose is considered a biodegradable, low-cost, useful, ecologically correct polymer that may be joined with other biomaterials to obtain novel characteristics and can, therefore, be used as a raw material to produce packaging. Bagasse, a waste rich in plant cellulose, can be reprocessed and used to produce and reinforce other materials. Based on these concepts, the aim of the current research was to design sustainable packaging material composed of bacterial cellulose (BC) and sugarcane bagasse (SCB), employing an innovative shredding and reconstitution method able to avoid biomass waste. This method enabled creating a uniform structure with a 0.10-cm constant thickness, classified as having high grammage. The developed materials, particularly the 0.7 BC/0.3 SCB [70% (*w*/*w*) BC plus 30% (*w*/*w*) SCB] composite, had considerable tensile strength (up to 46.22 MPa), which was nearly thrice that of SCB alone (17.43 MPa). Additionally, the sorption index of the 0.7 BC/0.3 SCB composite (235.85 ± 31.29 s) was approximately 300-times higher than that of SCB (0.78 ± 0.09 s). The packaging material was also submitted to other analytical tests to determine its physical and chemical characteristics, which indicated that it has excellent flexibility and can be folded 100 times without tearing. Its surface was explored via scanning electron microscopy, which revealed the presence of fibers measuring 83.18 nm in diameter (BC). Greater adherence after the reconstitution process and even a uniform distribution of SCB fibers in the BC matrix were observed, resulting in greater tear resistance than SCB in its pure form. The results demonstrated that the composite formed by BC and SCB is promising as a raw material for sustainable packaging, due to its resistance and uniformity.

## 1. Introduction

The demand for renewable carbon materials has increased in response to several global issues, such as environmental pollution, natural resource depletion, and global warming [1]. Such renewable sources are intended to decrease the production and utilization of unbiodegradable products that rely on finite fossil fuel resources [2]. Driven by resource scarcity, enhanced consumers’ consciousness, and strong commitment from governmental and nongovernmental organizations as well as academic and industrial communities, investigation into ecofriendly materials has recently shown remarkable expansion [3]. These sources should help regenerate natural assets, remain in use at maximum level for a long period, and reallocate key resources.

Papermaking is among the most polluting sectors [4]. The production of paper and cellulose involves the generation of waste that contains highly complex organic substances and inorganic pollutants from wood decomposition and cellulose chemical treatment. Therefore, this industry is classified as the sixth most polluting sector globally [4,5,6]. Plant cellulose (PC) is the most important feedstock for papermaking due to its abundance in nature and diverse applications. Plant cell wall is composed of approximately 50% cellulose, synthesized by means of a bioprocess sustained by solar energy [5]. Nonetheless, PC preparation and utilization raise various concerns. The amount of cellulose necessary to meet demands in the paper and derivatives industry brings with it a notable consumption of vegetation, mainly eucalyptus (*Eucalyptus globulus*), in addition to the chemicals needed for pulping and pulp bleaching. The wastewater generated in these processes is one of the main polluting sources, and its large-scale treatment involves significant electrical energy consumption (around 1000 kWh/ton). Thus, industries in all fields are interested in ecologically correct alternatives that minimize environmental harm [7]. Additionally, the intensive use of packaging results in massive waste production, predominantly from nonbiodegradable polymers that are difficult to recycle, exacerbating the waste problem [8].

In the search for solutions to this problem, the conversion of sugarcane (*Saccharum officinarum*) into alcohol and sugar results in agricultural waste, such as bagasse, which is generally discarded. However, it has a significant amount of PC, which may be reprocessed and used to prepare diverse materials, including paper and packaging, or as reinforcement material [9,10]. 

Despite plants being the main cellulose sources in the world, different types of microorganisms can also produce bacterial cellulose (BC) [11], which was first reported at the end of the 19th century [12]. It is a linear exo-polysaccharide or biofilm synthesized by some bacterial species that belong to *Achromobacter*, *Agrobacterium*, and *Acetobacter* genera among others. By employing a bacteria/yeast co-culture at temperatures around 25–30 °C and pH of approximately 4.5–7.5, a resistant nonwoven material was created with customizable features for specific uses [13,14]. Since BC is a cheap and very useful biopolymer, its various applications have increasingly been studied over the years. Even though BC has the same empirical and molecular formulae as PC, its fibrillar structure is smaller and its surface area larger. PC fibers have in fact a diameter varying from 13 to 22 μm and crystallinity between 44 and 65%, while BC fibrils are nano-scale in size (10–100 nm) and crystallinity of almost 90% [15,16,17]. Furthermore, BC is highly pure because its production is not associated with other constituents, like lignin, pectin and hemicellulose found in PC, thus avoiding the need for an environmentally unfriendly purification process [18,19].

In BC biosynthesis, many factors should be taken into consideration for controlling its quality, including microbial species, fermentation media, operating conditions, fermenters, recovery and downstream processes [20]. Microorganisms are initially grown on adequate culture media usually comprising carbon and nitrogen sources as well as essential micronutrients [21]. Fermentation media employed to produce BC are also pivotal, because they must guarantee a suitable environment for both microbial proliferation and cellulose production. Media composition can be tuned to improve BC synthesis through the optimization of operating parameters such as carbon/nitrogen ratio, pH, temperature, among others. Particularly, the carbon source has the function of supporting cell growth and BC production, and the cultivation must be kept under controlled conditions of temperature, pH, and oxygen level. Media nutrients and oxygen avoid the aggregation of BC fibers and ensure their even development. In such an environment, BC is produced by extrusion of cellulose chains through the external membrane and into the surroundings [22,23] and their subsequent alignment and interweaving, thus leading to a three-dimensional cellulose-fiber network.

The array of biocellulose use can be widened through modifications, and several studies were performed for functionalizing and using it as packaging material or in novel applications in medical, biomedical, electronics, and environmental fields [15,16]. Furthermore, biocellulose normally exploits widely available, low-cost sugars such as glucose or fructose, thereby making the carbon footprint and resource demand of the process very sustainable [24,25].

The characteristics of microbial cellulose make it an excellent basis for the production of sustainable packaging material. This material can, in fact, be prepared with variable shape and thickness, adapting itself to the format of vessel used for cultivation, which avoids losses in the assembly of parts. Small pieces or patches of BC can also be used through the homogenization and remodeling process [26,27]. The production of microbial cellulose can also benefit from the use of agro-industrial waste products, which reduces its cost and makes the product more accessible. Another advantage is that BC is easily biodegradable when discarded in nature [28]. However, despite these advantages, the large-scale production of BC faces technical and economic challenges, such as the need to optimize fermentation processes and significant energy requirements [17]. 

Sugarcane bagasse (SCB) was also proposed as an option for the manufacture of sustainable packaging, which can reduce the quantity of industrial wastes and promote the use of renewable resources. Despite the difficulties faced in the use of BC and sugarcane bagasse, it is encouraging to see that both options are being explored as more sustainable solutions in the packaging industry. Further studies and investments in technology could enable overcoming these challenges and promote the transition to more ecologically friendly packaging, thereby contributing to the preservation of the environment [29,30]. 

As Brazil is the largest global sugarcane producing country, with a harvest of 654.5 million tons in the 2020/21 season destined to produce more than 40 million tons of sugar and almost 30 million m^3^ of bioethanol [31], the present study was based on concepts, trends, and perspectives of microbial cellulose in the industry. The goal was to develop a packaging material based on both SCB and BC, employing an innovative shredding and reconstitution method to prepare a composite of uniform thickness to be used as a packaging material and in papermaking, while meeting the needs of increasingly conscious consumers and enabling the creation of novel products.

Research has shown that using BC as an additive can increase the strength of papers made from secondary fiber and agro-residues. Given this, our current study is centered on using BC to enhance the strength of packaging papers. A transdisciplinary approach with novel innovations can foster the development of new BC-based composites for broadening the arrays of applications. 

Sustainability requires a delicate balance among environmental, economic, and social issues. BC can be considered sustainable regarding supply of materials, water and energy utilization, and production of wastes. Additionally, one should consider the sustainability of products, workforce, and technological development. Thus, this study addresses a critical gap in sustainable packaging research by focusing on the integration of BC and SCB for preparing a new, eco-friendly biomaterial. The significance lies in its potential to reduce reliance on nonbiodegradable polymers, mitigate waste, and utilize abundant agricultural byproducts. The innovative shredding and reconstitution method employed not only enhances material properties but also promotes uniformity and high tensile strength. By advancing the development of sustainable packaging solutions, this research contributes to environmental preservation and meets the growing consumer demand for eco-conscious products.

## 2. Materials and Methods

### 2.1. Microorganisms and Maintenance

Microbial species naturally present in the Symbiotic Culture of Bacteria and Yeast (SCOBY) were employed to produce BC. Such a symbiotic culture, commonly called “kombucha”, is similar to mother of vinegar and tends to form a zoogleal mat known as a “mother”. The culture medium consisted of 10 g/L green tea leaves (*Camellia sinensis*), 50 g/L sucrose, and 1.15 g/L citric acid with pH adjusted to 5.5, according to Silva Junior et al. [28].

### 2.2. Culture Conditions as Well as Biocellulose Purification and Recovery

The production of BC involved pre-inoculation of the above consortium (10% *v*/*v*) into 2.5-L Schott flasks filled with 2.0 L of culture medium. The cultivation was conducted under static conditions at a temperature of 30 °C for 14 days. BC was washed with tap water purified by immersing it into a 0.1 M NaOH solution at 70 °C for 1 h. After weighing samples, the BC yield was calculated, according to Silva Junior et al. [28], as the BC mass produced per liter of fermentation broth and expressed in g/L.

### 2.3. Water Retention Capacity

The moisture content of any material is closely related to its water retention capacity (*WRC*). The water absorption and permeability capabilities are assessed through this test. BC samples were weighed and subjected to oven drying until a constant weight was achieved, allowing for complete removal of water. After determination of BC yield, *WRC* was calculated according to Medeiros et al. [32] using the equation:

(1) $$WRC\;\left(\%\right)=\frac{Mean\;wet\;mass\;\left(g\right)-Mean\;dry\;mass\;\left(g\right)}{Mean\;wet\;mass\;\left(g\right)}\times 100\;(\%)$$

### 2.4. Treatment of Sugarcane Bagasse 

SCB was submitted to a purification and dehydration process to eliminate undesirable substances. For this purpose, bagasse was shredded in a knife mill until reaching a fibrous consistency and then dried for 12 h using a forced-air circulating system operating at 60 °C. The dried SCB was added to a 3.0% NaOH solution that was reutilized from the BC purification process and maintained at 80 °C for 2 h to allow the complete removal of lignin and other impurities. After this step, the material was neutralized (pH 7.0) and kept submerged in deionized water. 

### 2.5. Production of Sugarcane Bagasse/Biocellulose Packaging Material

**Figure 1:**
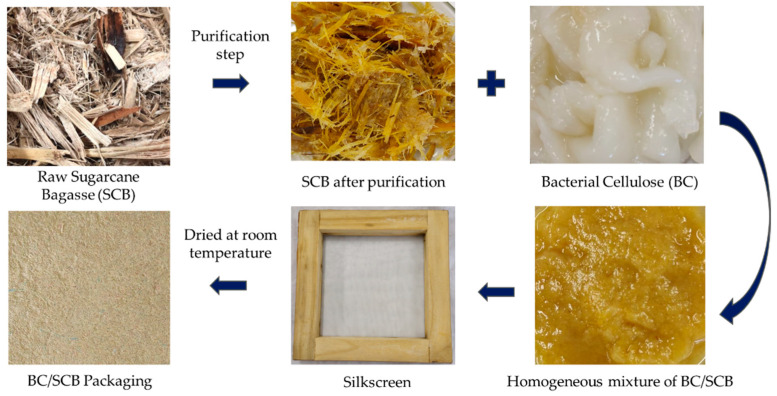
Scheme of production of bacterial cellulose (BC) and sugarcane bagasse (SCB) packaging material.

To prepare SCB/BC packaging material, we prepared BC-SCB mixtures in two different proportions, namely 50% (*w*/*w*) BC plus 50% SCB (*w*/*w*) and 70% (*w*/*w*) BC plus 30% SCB, which will be called from now onwards as BC/SCB and 0.7 BC/0.3 SCB, respectively. Purified BC was shredded in an industrial shredder operating at 18,000 rpm for 2 min for obtaining a homogeneous sample. The treated bagasse was then added, followed by mixing for another 2 min until the material acquired a uniform consistency. The material was then carefully spread over a silkscreen (20 cm × 20 cm), enabling its reconstitution, and the mixture was allowed to completely dry at room temperature for 24 to 48 h. The process, which was considered concluded only after the complete reconstitution of the material, is outlined in Figure 1.

### 2.6. Water Contact Angle and Sorption Index Assessment

**Figure 2:**
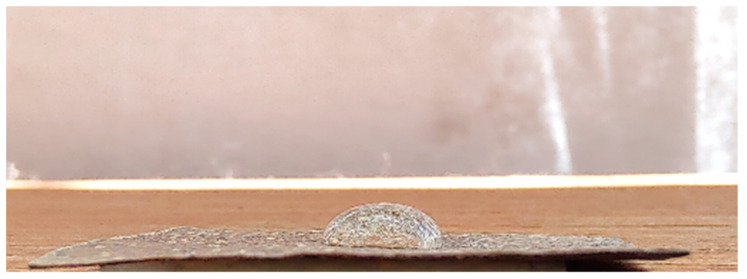
Water drop deposited on the surface of the SCB sample.

To carry out this analysis, samples were prepared in a rectangular format measuring 10 mm × 5 mm. The water contact angle (*WCA*) was assessed by observing water-sample interactions. Thus, samples were placed on a flat surface, and a goniometer and a mirrorless digital camera (XT10, Fujifilm, Minato City, Japan) were used. According to the sessile drop procedure, a drop was placed on sample membrane surface (Figure 2), and *WCA* was measured after 1 s. For determination of sorption index (*SI*) (s), the drop was examined for up to 10 min or till absorption was completed, and the mean time (s) was recorded [33]. Samples were tested at room temperature (25 ± 1 °C) under standard relative humidity conditions for Brazil (around 60%).

### 2.7. Swelling Ratio

The water absorption rate is crucial in sustainable packaging due to its influence on durability, protection against contamination, structural stability, and minimization of chemical additives, contributing to the overall functionality and sustainability of packaging. The samples used for this test had the same shape (rectangular) and size (10 mm × 5 mm) as in the previous one, and had their residual moisture removed in an oven at 50 °C until they reached a constant weight. Next, samples were weighed and dipped in distilled water at room temperature for 24 h. They were then recovered, had their excess surface water removed, and were weighed again. The swelling ratio (*SR*) was assessed as the mass variation before and after swelling using the relationship:

(2) $$SR=\frac{Swollen\;mass\;\left(g\right)-Starting\;mass\;(g)}{Starting\;mass\;(g)}$$

### 2.8. Fourier Transform Infrared Spectroscopy

Specimens were first left in a desiccator containing silica gel at room temperature. The analysis was carried out on a Fourier transform infrared (FTIR) spectrometer (Alpha II, Bruker Co., Ettlingen, Germany) equipped with a horizontal attenuated total reflectance accessory using a crystal cell plate (ZnSe 45°; size: 80 × 10 mm; thickness: 4 mm) (PIKE Technology Inc., Madison, WI, USA). The functional groups in specimens were singled out after 32 scans at 4 cm^−1^ resolution in the wavenumber range from 4000 to 400 cm^−1^.

### 2.9. Thermogravimetry

To determine the thermostability of specimens, thermogravimetric analysis (TGA) was carried out using a simultaneous thermal analyzer (TGA-50, Shimadzu, Kyoto, Japan) with a platinum support for each sample, the mass of which was approximately 10 mg. Readings were conducted in the temperature range 25–800 °C, at 10 °C/min heating rate and 50 mL/min nitrogen flowrate. TGA findings were illustrated in curves that showed the change in mass versus temperature, enabling the determination of specimen composition and thermal stability as well as other relevant information.

### 2.10. Scanning Electron Microscopy Coupled to Energy Dispersive Spectroscopy

Specimens were placed on copper stubs using dual adhesive carbon conductive tape and coated with gold for 30 s (SC-701 Quick Coater, Sanyu Denshi, Tokyo, Japan). Elements in specimens were qualitatively identified on an energy dispersive spectroscope (EDS) with 0.1% mass concentration detection limit coupled with a scanning electron microscope (SEM) (Inspect S50, FEI Company, Hillsboro, OR, USA). Fiber size was measured using the image-processing software ImageJ (NIH, Bethesda, MD, USA).

### 2.11. Flexibility

To determine the flexibility of the materials developed, samples were folded 100 times by hand, and flexibility was determined as the number of times the sample could be folded before tearing. The flexibility was classified, according to Chen et al. [34], according to the number of bends: poor (<20), fair (20–49), good (50–99) and excellent (>100). The samples were tested at room temperature and under standard relative humidity conditions for Brazil (around 60%). 

### 2.12. Mechanical Tests

The following aspects were investigated to assess the mechanical features of the materials developed: tensile strength to the point of tearing (N), maximum deformation (%), and Young’s modulus (MPa). The samples were cut into five rectangular strips measuring 2.0 × 7.0 cm. The tensile strength test was conducted at room temperature and a rate of 0.5 m/min using a 1 kN load cell in a universal testing machine (Instron 5969, Norwood, MA, USA), following the ASTM D882 method. 

### 2.13. Statistical Analyses

The results were submitted to analysis of variance (ANOVA) with the Statistica^®^ (version 7.0) program. Data from triplicate experiments were expressed as mean ± standard deviation (SD), with 95% confidence interval corresponding to 5% significance level (*p* < 0.05).

## 3. Results and Discussion

### 3.1. Biocellulose Yield and Water Retention Capacity

The average yield of wet BC was 450.49 ± 16.61 g/L of medium and that of dry BC 13.41 ± 1.17 g/L after 14 days of fermentation. These values are quite promising compared to findings described in other studies. For instance, under static conditions Revin et al. [35] found dry BC yields of 9.5 ± 0.1 and 9.2 ± 0.1 g/L after 14 days of culture using *Komagatabacter sucrofermentans* H 110 and *Komagatabacter hansenii* C 110, respectively, while Silva Junior et al. [28] found a hydrated BC yield of 422.12 ± 15.26 g/L after the same period of time.

**Figure 3:**
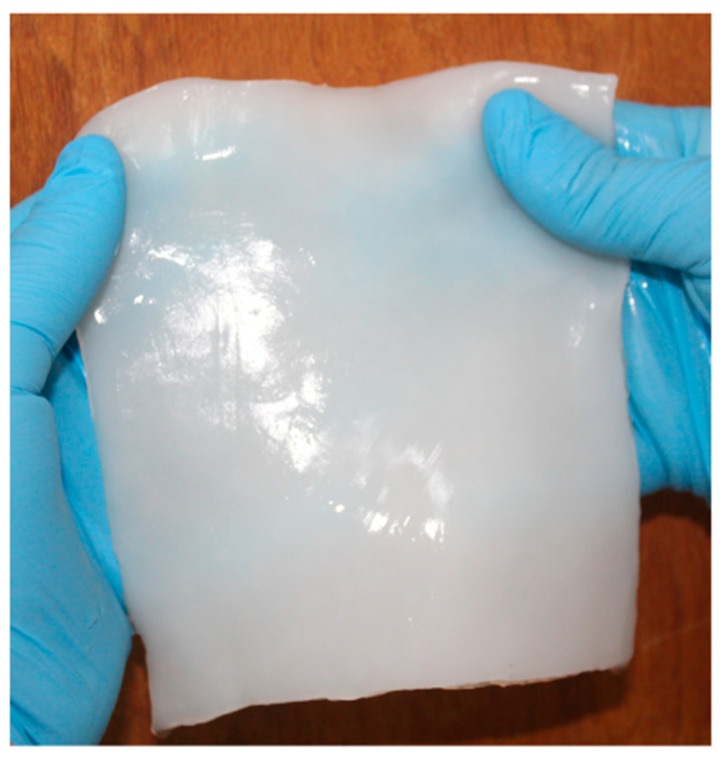
Appearance of biocellulose after the purification stage.

The purification stage using NaOH favored a uniform color along with the removal of compounds and debris of the fermented broth possibly adsorbed onto the BC surface (Figure 3). 

The data listed in Table 1 highlight the high *WRC* of BC (>97%), which is close to values reported by Silva Junior et al. [28], Costa et al. [36], and Nascimento et al. [37]. This is one of the fundamental features of novel BC-based functional materials, as it enables them to arrange in a three-dimensional structure, giving them unique physical characteristics such as notable porosity and flexibility. Moreover, water plays a pivotal role in elasticity and strength, filling spaces among cellulose fibers and giving the fibers the ability to absorb water and swell without compromising structural integrity, which makes BC useful in applications requiring strong, flexible materials. The presence of water also simplifies the BC modification proposed in the present study, enabling the incorporation of additives by taking advantage of water properties to adjust BC characteristics in accordance with specific needs, such as its use as packaging with the addition of SCB.

### 3.2. SCB/BC Packaging Material Production 

As explained in Section 2.5, purified membranes were shredded together with SCB using two different proportions, namely 50% (*w*/*w*) BC plus 50% SCB (*w*/*w*) (BC/SCB) and 70% (*w*/*w*) BC plus 30% SCB (0.7 BC/0.3 SCB). The mixture was distributed on a surface, spread evenly, and set to dry, enabling the reconstitution of the fiber arrangement during the process. The sheets resulting from the reconstitution had visual characteristics resembling paper. 

**Figure 4:**
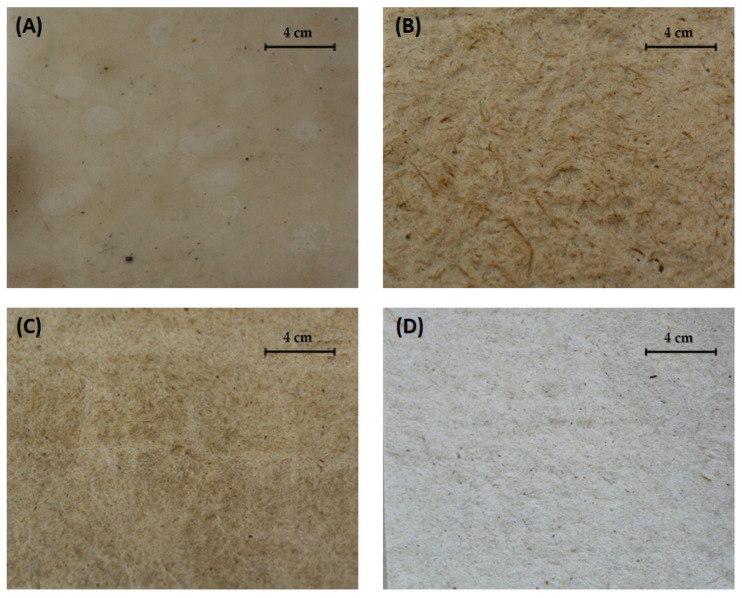
Visual aspect of samples of bacterial cellulose (BC) and sugarcane (SCB) mixed in different proportions. (**A**) BC alone, (**B**) SCB alone, (**C**) 50% (*w*/*w*) BC plus 50% (*w*/*w*) SCB, and (**D**) 70% (*w*/*w*) BC plus 30% (*w*/*w*) SCB.

Pure BC had an appearance similar to leather, with a whitish color. The other samples were similar and had visual and textural aspects resembling recycled paper, with different tones depending on the proportions of the materials used in the membrane formulation (Figure 4). Although the successful production of BC-SBC paper sheets is unique in the literature, other BC-based composites displayed characteristics demonstrating that BC can be used in other forms to be applied as packaging. Zhou et al. [38] produced novel, ecological films for food packaging using curdlan, BC, and cinnamon essential oil. 

The development of microbial cellulose-based packaging material was successful, resulting in a material similar to paper with 0.10-cm constant thickness and high grammage (about 255 g/m^2^). Moreover, the process produced no residue, as all cellulose scraps could be shredded together to form a single membrane, which was molded and dried in the desired shape.

### 3.3. Water Contact Angle, Swelling Ratio, and Sorption Index 

Table 2 lists the values of *WCA*, *SR* and *SI* of tested samples (BC, SCB, BC/SCB, and 0.7 BC/0.3 SCB).

The BC sample had a *WCA* of 39.29°, which is indicative of moderate hydrophilicity and implies some degree of water repellency while not being highly water resistant [39]. On the other hand, its values of *SR* (2.79 ± 0.27) and *SI* (77.32 ± 3.12 s) demonstrate ability to absorb and retain water up to 2.79 times its initial weight and remarkable water-holding capacity, respectively.

In contrast, the SCB sample had a lower *WCA* (15.11°), indicating a higher degree of hydrophilicity compared to the BC membrane. This suggests that SCB had a greater propensity to absorb water than BC, which is confirmed by an approximately 70% higher *SR* value (4.77 ± 0.29). Nonetheless, its much lower *SI* value (0.78 ± 0.09 s) indicates that it had a small water-holding capacity compared to BC.

The BC/SCB composite had a higher *WCA* (48.60°), suggesting a more hydrophobic surface compared to both BC and SCB. The *SR* of this composite was 2.16 ± 0.14, indicating moderate water absorption capacity (between that of BC and SCB). Notably, its *SI* was higher (141.14 ± 22.13 s), demonstrating a significantly higher water-holding capacity over time than BC and SCB, likely due to a synergistic action of these components.

Finally, the 0.7 BC/0.3 SCB composite had the highest *WCA* (53.22°), indicating a more hydrophobic nature. Despite having the lowest *SR* (1.57 ± 0.3) among the samples, it showed the highest *SI* (235.85 ± 31.29 s), implying the highest water retention capacity over time. 

These findings are of considerable significance for understanding and tailoring the performance of these materials, particularly for applications in which the materials come into contact with liquids, such as packaging materials [40].

### 3.4. Fourier Transform Infrared Spectroscopy

**Figure 5:**
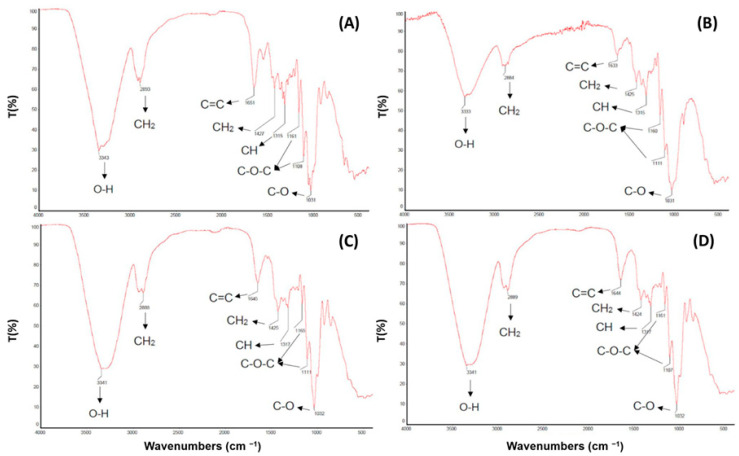
FTIR spectra of bacterial cellulose (BC) and sugarcane bagasse (SCB) mixed in different proportions. (**A**) BC alone, (**B**) SCB alone, (**C**) 50% (*w*/*w*) BC plus 50% (*w*/*w*) SCB, and (**D**) 70% BC plus 30% (*w*/*w*) SCB.

Figure 5 illustrates the FTIR spectra of BC, SCB and both composite materials (BC/SCB and 0.7 BC/0.3 SCB), which allowed us to identify their main functional groups (Table 3), including those likely responsible for previous observations.

BC and SCB had similar characteristic peaks, including those assigned to the axial deformations of O-H and CH_2_ bonds and deformation of C=C bonds, consistently with the fact that they share common functional groups, as shown in Table 3, due to the presence of cellulose-based compounds in their structures [41].

Even the BC/SCB and 0.7 BC/0.3 SCB composites displayed characteristic peaks similar to those found in BC and SCB alone, indicating the presence of hydroxyl groups, aliphatic compounds, and unsaturated compounds [42].

Particularly, the wide band in the wavenumber range 3000–3500 cm^−1^ can be assigned to BC hydroxy groups involved either in intra- or inter-chain hydrogen bonding, whose intensity was related to the hydrophilicity of the material and its strong interaction with water [43]. 

The similarity in chemical characteristics suggests that both composites retained the chemical properties of both BC and SCB. The use of different ratios of these components slightly impacted the intensity or exact positions of their absorption peaks, but the fundamental functional groups remained the same.

### 3.5. Thermogravimetric Analysis

**Figure 6:**
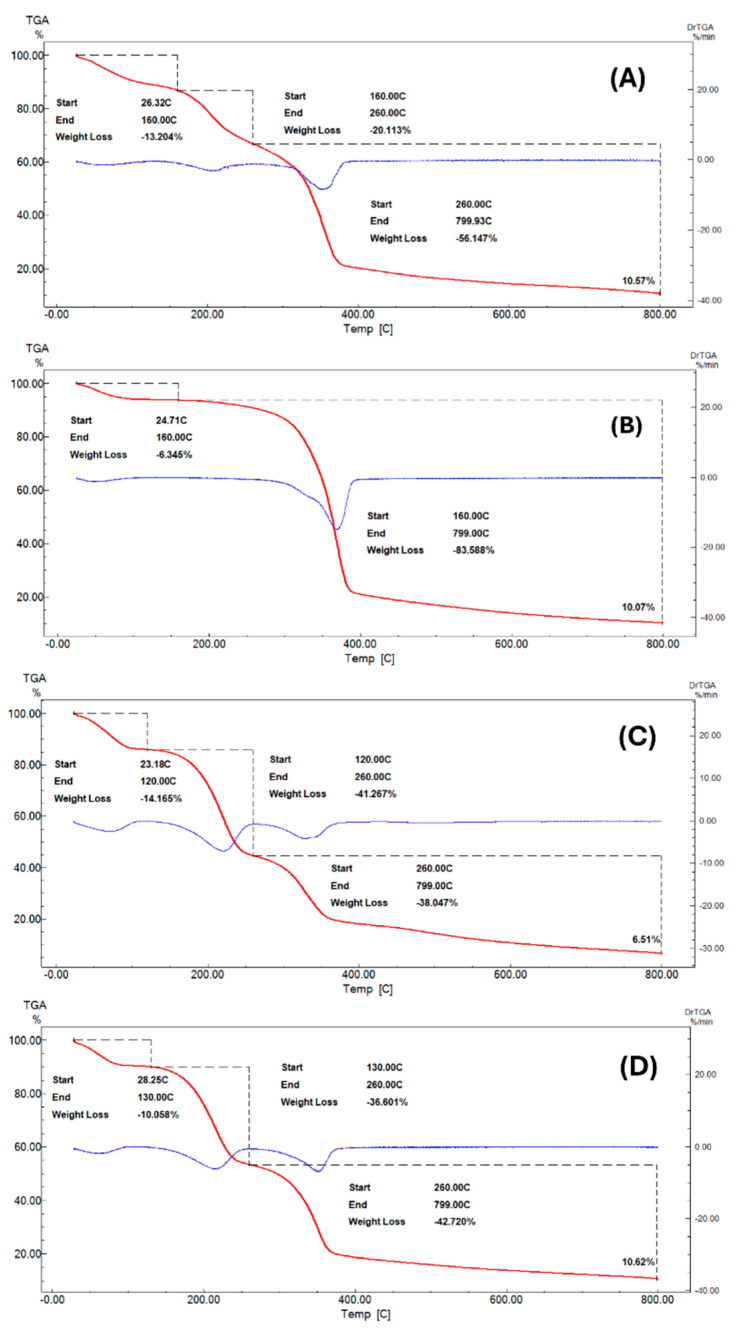
TG curves of bacterial cellulose (BC) and sugarcane bagasse (SCB) mixed in different proportions. (**A**) BC alone, (**B**) SCB alone, (**C**) 50% (*w*/*w*) BC plus 50% (*w*/*w*) SCB, and (**D**) 70% (*w*/*w*) BC plus 30% (*w*/*w*) SCB.

As shown by thermogravimetric analysis (TGA) (Figure 6) and related results (Table 4), all samples, with the exception of SCB, exhibited three distinct degradation events in the ranges of 25–160 °C, 130–160 °C, and 260–800 °C. The first event could be attributed to the loss of moisture and volatile constituents of samples, the second one to the decomposition of labile organic compounds, and the third and final event to the degradation of more stable organic components [44].

On the other hand, the SCB sample showed only two degradation temperature ranges, namely 25–160 °C and 160–800 °C. The lower range involved a lower mass loss (6.35%), suggesting loss of moisture and volatile constituents, as seen for BC. The higher range (160 to 800 °C) involved a notable mass loss (83.59%), which is indicative of extensive degradation of the SCB organic components.

### 3.6. Scanning Electron Microscopy (SEM) Coupled to Energy Dispersive Spectroscopy (EDS)

SEM was employed for comparing the morphologies of BC-membrane surfaces, investigating the adhesion of the plant cellulose fibers from SCB to the BC nanofibers, and examining the surface of the produced composites, which are all essential to establish the suitability of these mixtures as packaging materials. 

**Figure 7:**
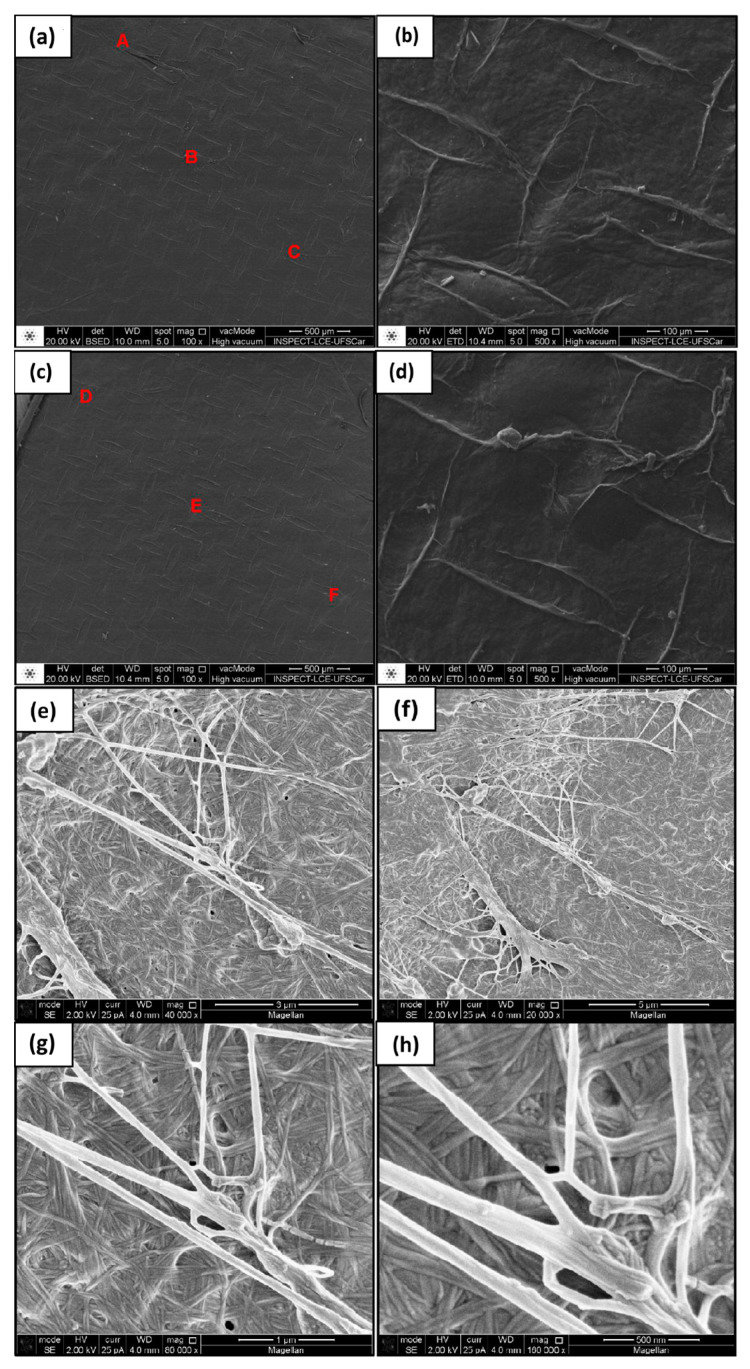
Micrographs of bacterial cellulose with magnification of (**a**) 100×, (**b**) 500×, (**c**) 100×, (**d**) 500×, (**e**) 20 k×, (**f**) 40 k×, (**g**) 80 k×, (**h**) 160 k×. Samples chosen for the EDS analysis were taken from points A, B, C, D, E and F.

At low magnification (100×), the BC membranes showed a uniform distribution of surface fibers without holes or unfilled areas (Figure 7a,c). An increase in magnification to 500× (Figure 7b,d) enabled us to validate the surface with saliences of the composing fibers, confirming good capacity for use as packaging [32,45,46].

Greater magnification in Figure 7g (80 k×) and Figure 7h (160 k×) shows that the secreted fibrils did not follow a specific pattern, were interlaced, and had a diameter of around 83.18 nm (measured using the ImageJ program) [47]. These organizational and size observations are considered to enable interlacing of fibers, which makes the material stronger and more capable of being mixed with other types of fibers to prepare novel biomaterials [48,49].

The results of the EDS listed in Table 5 demonstrate that the purification process and washing of the membranes were effective in removing microbial cells but not components of the culture medium [46,50,51]. The analysis revealed that the samples were composed of 94–97% carbon and oxygen, which are elements that make up cellulose, but considerable amounts of zinc, chlorine, potassium, and calcium were also present in practically all the samples. The presence of aluminium, magnesium, and silicon suggests the contamination of point B, which was the only sample containing these elements [51,52].

**Figure 8:**
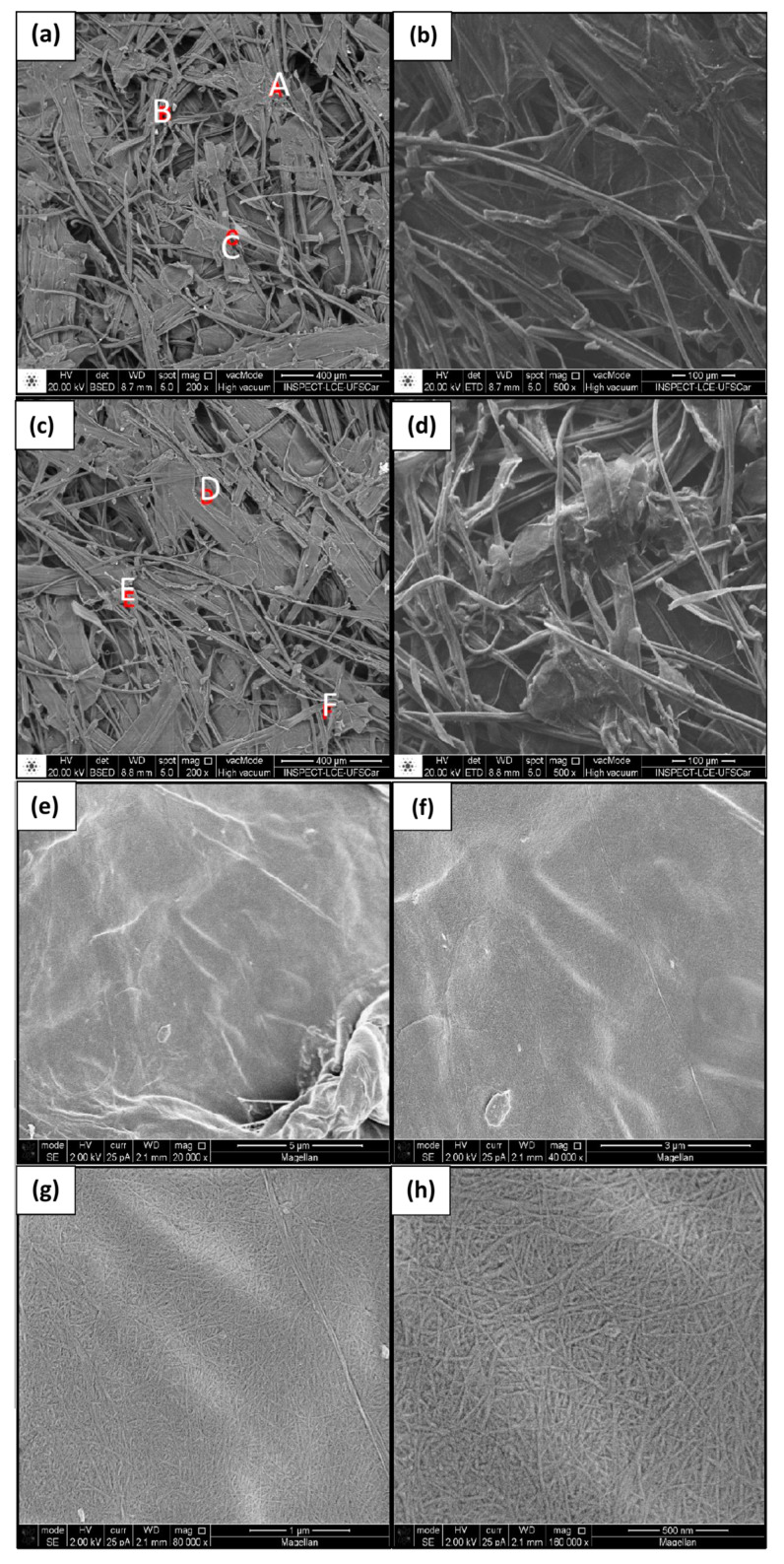
Micrographs of sugarcane bagasse at magnification of (**a**) 200×, (**b**) 500×, (**c**) 200×, (**d**) 500×, (**e**) 20 k×, (**f**) 40 k×, (**g**) 80 k×, (**h**) 160 k×. Samples chosen for the EDS analysis were taken from points A, B, C, D, E and F.

The microscopic examination of SCB showed some similar and some different characteristics from those found in BC membranes (Figure 8).

Particularly, the formation of fibers followed a different pattern, with larger, less organized fibers and more space among them. The heterogeneous surface seen at low magnification (Figure 8c,d) had a shape of the original plant fibers of the plant that indicates a complex structure and heterogeneity in components [53].

The results of the EDS analyses (Table 6) show the occurrence of elements that are not naturally present in the normal plant cellulose, suggesting that cutting, removal from the broth, and treatment of SCB left residues of other elements in samples [51].

**Figure 9:**
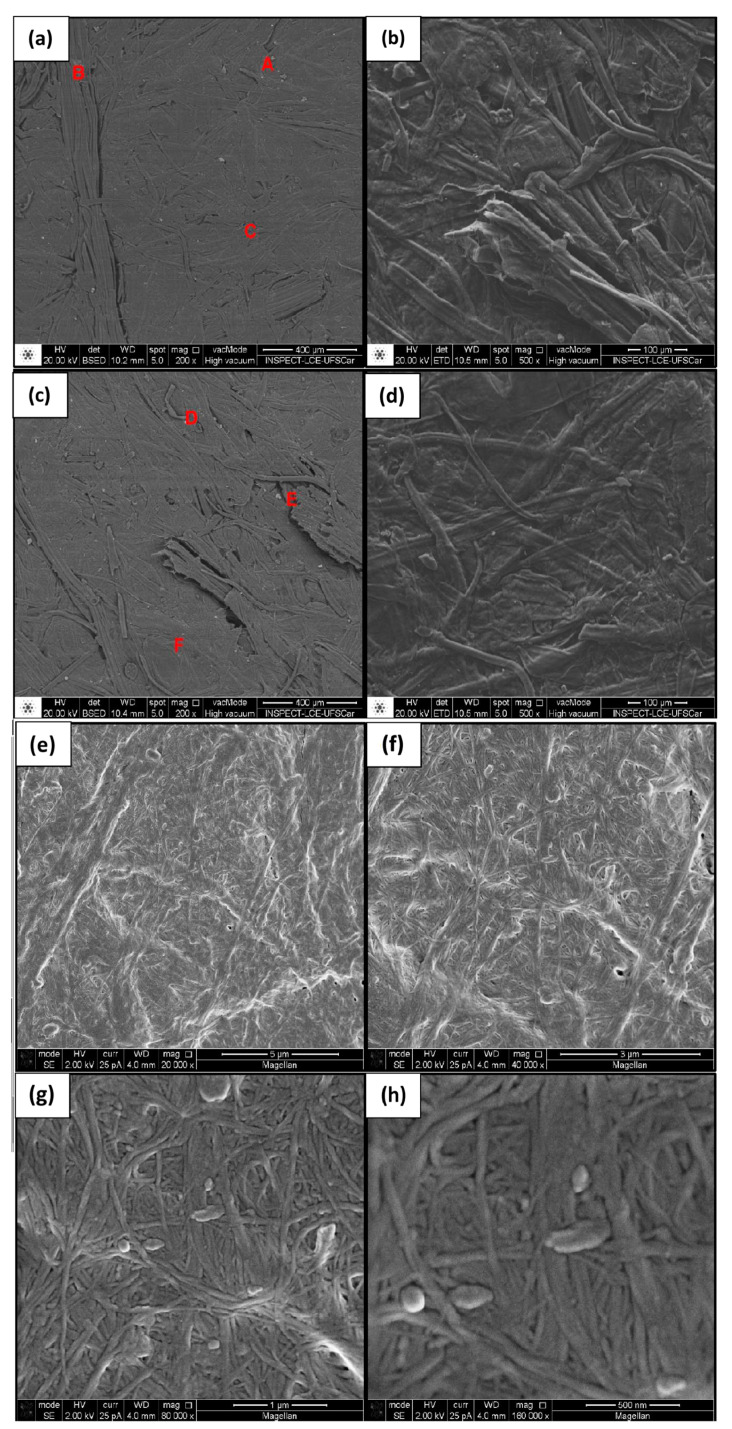
Micrographs of the BC/SCB composite, composed of 50% (*w*/*w*) bacterial cellulose plus 50% (*w*/*w*) sugarcane bagasse, with magnification of (**a**) 200×, (**b**) 500×, (**c**) 200×, (**d**) 500×, (**e**) 20 k×, (**f**) 40 k×, (**g**) 80 k×, (**h**) 160 k×. Samples chosen for the EDS analysis were taken from points A, B, C, D, E and F.

**Figure 10:**
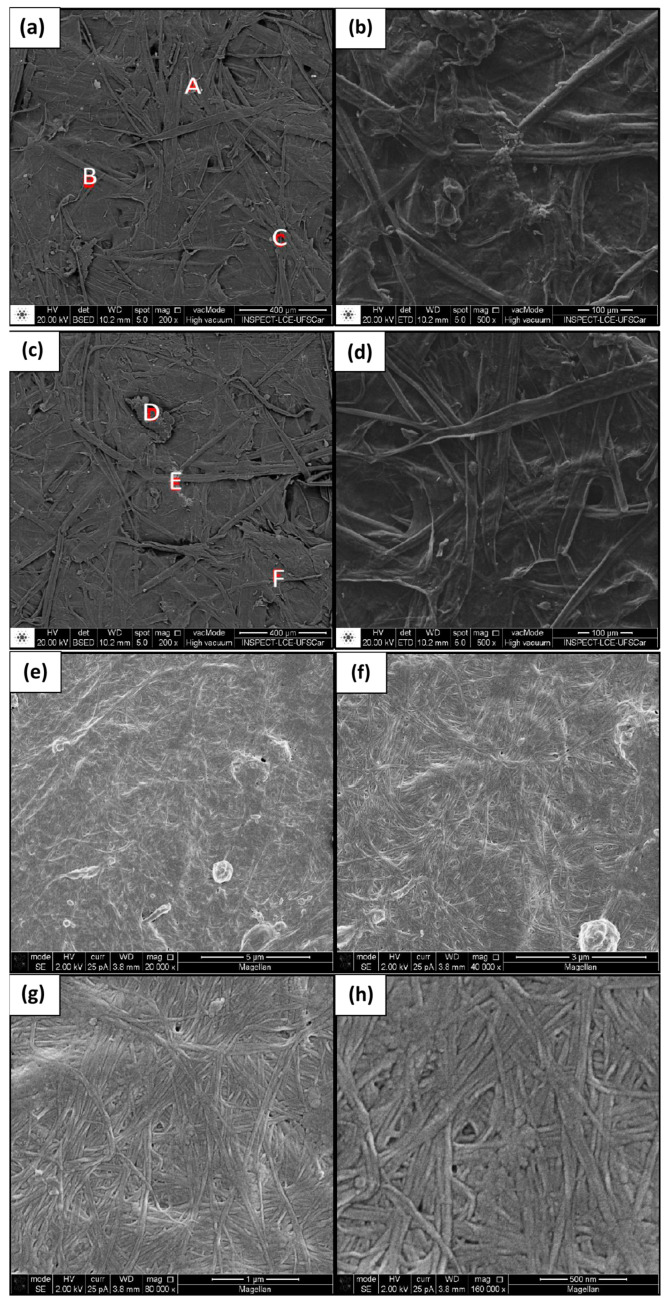
Micrographs of the 0.7 BC/0.3 SCB composite, composed of 70% (*w*/*w*) bacterial cellulose plus 30% (*w*/*w*) sugarcane bagasse, with magnification of (**a**) 200×, (**b**) 500×, (**c**) 200×, (**d**) 500×, (**e**) 20 k×, (**f**) 40 k×, (**g**) 80 k×, (**h**) 160 k×. Samples chosen for the EDS analysis were taken from points A, B, C, D, E and F.

Figure 9 and Figure 10 demonstrate that the two composites containing BC and SCB in different proportions had a uniform surface, suggesting a uniform distribution of SCB fibers in BC matrix. The mixture enabled better interlacing of the fibers, leading to a smoother surface, but with denser, more rigid fibers and greater filling of interstices. This was due to the fact that the BC and SCB fibers range in size, fitting into each other like pieces of a puzzle. 

This greater uniformity also suggests that samples underwent the reconstitution process, with a better texture in terms of smoothness, losing the initial SCB roughness. The reconstitution process is also expected to give the material better adhesion of fibers than SCB alone [46], with a direct influence on mechanical features.

Overall, these results demonstrate the achievement of the desired characteristics for composites prepared in the present study, i.e., the conversion of BC, originally leather-like in appearance, into a paper-like material, as well as that of SCB, of rougher quality, into a smoother material with a better visual quality for packaging applications. 

The EDS analysis (Table 7 and Table 8) showed that SCB elements remained even after shredding and reconstitution, indicating that they were closely linked to the structure of the SCB fibers. This was confirmed by the fact that the incidence of the elements diminished with the increase in the percentage of BC in the composites [32].

### 3.7. Flexibility and Mechanical Tests

The mechanical properties and flexibility of any packaging material impact its performance in processing and assembly, with features imposed by handling, storage, and distribution. So, evaluating the mechanical properties of packaging materials is of utmost importance, as they should keep their functionality during and after processing [54].

**Figure 11:**
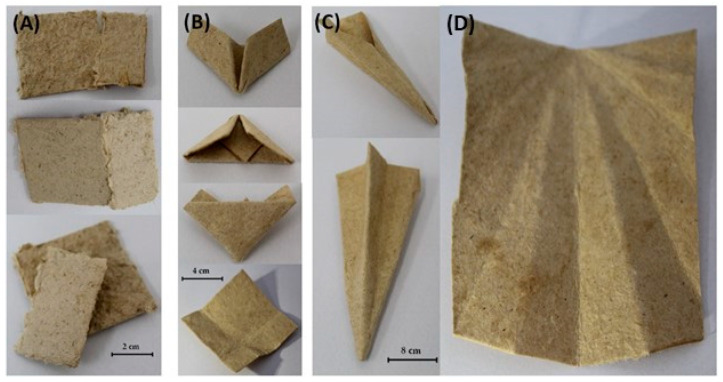
Appearance of bacterial cellulose (BC) and sugarcane bagasse (SCB) samples after undergoing flexibility tests. (**A**) SCB alone, (**B**) 50% (*w*/*w*) BC plus 50% (*w*/*w*) SCB, and (**C**,**D**) 70% BC plus 30% (*w*/*w*) SCB.

BC exhibited excellent flexibility, as shown in Table 9. The >100 score indicates that the BC sample is highly flexible and can withstand bending and deformation without tearing. The higher flexibility of BC (>100) compared to the other samples can be attributed to its nanofibrillar structure, which enables it to be molded into various shapes and configurations without losing its structural integrity [55]. In contrast, the lower flexibility score of SCB (only 26) (Figure 11A) may have been due to its fibrous, more rigid nature, which is typical of plant-based materials [56].

However, when BC and SCB were mixed, the resulting composites exhibited excellent flexibility, being able to be folded more than 100 times without tearing (Table 9). This indicates that the mixture of BC with SCB improved the flexibility of the material, enabling it to be folded several times and assembled into different shapes (Figure 11B–D), i.e., to meet essential specifications for the manufacture of resistant packages.

**Figure 12:**
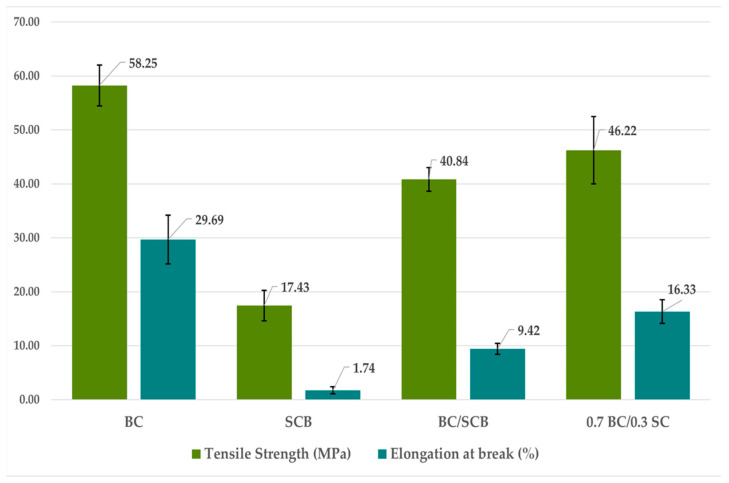
Graph of tensile strength (MPa) and elongation at break (%) of bacterial cellulose (BC) and sugarcane bagasse (SCB) samples. BC = BC alone, SCB = SCB alone, BC/SCB = 50% (*w*/*w*) BC plus 50% (*w*/*w*) SCB, 0.7 BC/0.3 SCB = 70% (*w*/*w*) BC plus 30% (*w*/*w*) SCB.

The results of mechanical tests (Figure 12) showed that BC alone was able to withstand a tensile stress before breaking (58.25 MPa) more than three times that of SCB (17.43 MPa), and to be stretched 29.69% more than its initial length prior to breaking, compared to only 1.74% found for SCB. So, SCB alone, being more easily torn, was less flexible and resistant than BC, making it inappropriate as a strong, functional packaging.

In contrast, a significant enhancement in mechanical features was found in the mixtures of BC and SCB. Besides the flexibility test, which demonstrated resistance to successive folding along the same line, composites were able to withstand tensile stress of 40.84 MPa (BC/SCB) and 46.22 MPa (0.7 BC/0.3 SCB) prior to breaking, which is, as an average, 2.5-times more than that withstood by pure SCB (17.43 MPa). Moreover, these composites were able to be stretched five and nine times more prior to tearing. This indicates that BC incorporation improved the biomaterial mechanical features for the desired application, enabling the original material, which previously tore with greater ease, to become stronger and, consequently, more versatile.

These improved characteristics were also evident in the SEM images of the BC/SCB and 0.7 BC/0.3 SCB composites (Figure 9 and Figure 10), which highlighted that their surface had a greater filling of spaces, indicating greater adhesion of fibers and, consequently, greater tear resistance than SCB in its pure form.

## 4. Conclusions

The results of this study demonstrate the versatility of bacterial cellulose as a potential platform for customizing materials. Researchers have been working on chemical modifications, physical enhancements, and incorporating other materials to improve its mechanical, thermal, and biological properties. These strategies aim to broaden the array of possible applications and highlight the ability of the resulting composites to meet the needs of various industries. Importantly, the biodegradability and biocompatibility of bacterial cellulose align with the increasing focus on sustainable and eco-friendly materials. The use of biomaterials as packaging materials, made from BC membranes reinforced with SCB, offers new prospects in the market, attracting attention in all steps of the process, from production to consumption. The growing interest in these materials promises to optimize fabrication processes, making the production of sustainable materials more accessible and economically viable, while also adding scientific and technological value and contributing to environment protection. The method described in this study resulted in packaging materials in which flexibility, mechanical strength, and visual aspects were improved by mixing BC with SCB. However, additional efforts are required to enhance the uniformity of these composites as well as investigate other proportions of BC and SCB, other forms of production and drying, and other additives that can enable novel applications. Future prospects for developed biomaterials are encouraging. As research in this field continues to unfold, the material’s potential to reshape industries and contribute to sustainable technological advancements is exciting. The current results highlight the potential of sustainable packaging based on BC reinforced with SCB as an eco-friendlier option for an increasingly conscious market and consumer population, in addition to demonstrating the possibility, after the new tests described above, of large-scale production.

## Figures and Tables

**Table 1:** Yield and water retention capacity (*WRC*) of bacterial cellulose (BC) produced after 14 days of culture. Results, expressed as mean ± SD (n = 3), were significant at *p* < 0.05.

Weight Basis	Yield (g/L)	*WRC* (%)
Wet	450.49 ± 16.61	97.02 ± 0.23
Dry	13.41 ± 1.17 g/L	

**Table 2:** Values of water contact angle, swelling ratio and sorption index of the different samples prepared. BC = Bacterial cellulose; SCB = Sugarcane bagasse; BC/SCB = 50% (*w*/*w*) BC plus 50% (*w*/*w*) SCB; 0.7 BC/0.3 SCB = 70% (*w*/*w*) BC plus 30% (*w*/*w*) SCB. Results, expressed as mean ± SD (n = 3), were significant at *p* < 0.05.

Sample	Water Contact Angle (°)	Swelling Ratio	Sorption Index (s)
BC	39.29	2.79 ± 0.27	77.32 ± 3.12
SCB	15.11	4.77 ± 0.29	0.78 ± 0.09
BC/SCB	48.60	2.16 ± 0.14	141.14 ± 22.13
0.7 BC/0.3 SCB	53.22	1.57 ± 0.3	235.85 ± 31.29

**Table 3:** Main functional groups detected from FTIR spectra of bacterial cellulose (BC) and sugarcane bagasse (SCB) mixed in different proportions. BC = BC alone, SCB = SCB alone, BC/SCB = 50% (*w*/*w*) BC plus 50% (*w*/*w*) SCB, and 0.7 BC/0.3 SCB = 70% BC plus 30% (*w*/*w*) SCB.

Sample	Wavenumber (cm^−1^)	Characteristic Peaks
BC	3343	Axial deformation of O-H
	2893	Axial deformation of CH_2_
	1636	Deformation of C=C
	1427	Angular deformation of CH_2_
	1314	Angular deformation of CH
	1161	Stretching of C-O-C
	1108	Stretching of C-O-C
	1032	Deformation of C-O
SCB	3333	Axial deformation of O-H
	2884	Axial deformation of CH_2_
	1633	Deformation of C=C
	1425	Angular deformation of CH_2_
	1315	Angular deformation of CH
	1160	Stretching of C-O-C
	1111	Stretching of C-O-C
	1031	Deformation of C-O
BC/SCB	3341	Axial deformation of O-H
	2888	Axial deformation of CH_2_
	1645	Deformation of C=C
	1425	Angular deformation of CH_2_
	1317	Angular deformation of CH
	1165	Stretching of C-O-C
	1111	Stretching of C-O-C
	1032	Deformation of C-O
0.7 BC/0.3 SCB	3341	Axial deformation of O-H
	2889	Axial deformation of CH_2_
	1644	Deformation of C=C
	1424	Angular deformation of CH_2_
	1317	Angular deformation of CH
	1161	Stretching of C-O-C
	1107	Stretching of C-O-C
	1032	Deformation of C-O

**Table 4:** TGA results for bacterial cellulose (BC) and sugarcane bagasse (SCB) mixed in different proportions. BC = BC alone, SCB = SCB alone, BC/SCB = 50% (*w*/*w*) BC plus 50% (*w*/*w*) SCB, and 0.7 BC/0.3 SCB = 70% BC plus 30% (*w*/*w*) SCB.

Sample	Temperature Range (°C)	Loss of Mass (%)	Residue at 800 °C (%)
BC	26–160	13.20	10.57
	160–260	20.11	
	260–800	56.15	
SCB	25–160	6.35	10.07
	160–800	83.59	
BC/SCB	23–120	14.17	6.51
	120–260	41.27	
	260–800	38.05	
0.7 BC/0.3 SCB	28–130	10.06	10.62
	130–260	36.60	
	260–800	42.72	

**Table 5:** Results of EDS analysis of bacterial cellulose (BC). Letters A, B, C, D, E and F refer to points (Figure 7) where samples were taken for the EDS analysis.

Element	Mass Percentage (%)						
	A	B	C	D	E	F	Average Value
C	46.39	47.54	48.96	48.11	48.31	48.37	47.95 ± 0.89
O	50.15	46.97	46.95	50.33	48.35	48.13	48.65 ± 1.49
Zn	1.57	1.37	1.78	-	1.40	1.32	1.49 ± 0.19
Mg	-	0.37	-	-	-	-	0.37
Al	-	0.39	-	-	-	-	0.39
Si	-	0.53	0.26	-	-	-	0.39 ± 0.19
Cl	0.81	1.13	1.18	0.84	0.80	1.04	0.97 ± 0.17
K	0.51	0.48	-	-	0.39	0.45	0.46 ± 0.05
Ca	0.57	1.22	0.87	0.72	0.74	0.69	0.80 ± 0.23

**Table 6:** Results of EDS analysis of sugarcane bagasse (SCB). Letters A, B, C, D, E and F refer to points (Figure 8) where samples were taken for the EDS analysis.

Element	Mass Percentage (%)						
	A	B	C	D	E	F	Average Value
C	48.70	46.85	48.32	48.43	48.52	48.71	48.25 ± 0.71
O	47.21	47.65	48.11	47.69	48.20	48.29	47.85 ± 0.42
Zn	0.67	0.78	0.65	0.85	0.87	0.50	0.71 ± 0.14
Mg	0.28	0.33	0.32	-	-	-	0.31 ± 0.04
Si	0.40	0.44	0.30	0.34	0.49	0.22	0.36 ± 0.10
Cl	0.46	0.48	0.53	0.52	-	0.74	0.55 ± 0.11
K	-	1.62	0.37	-	-	0.31	0.76 ± 0.72
Ca	2.28	1.85	1.39	2.17	1.92	1.24	1.81 ± 0.42

**Table 7:** Results of EDS analysis of the BC/SCB composite composed of 50% (*w*/*w*) bacterial cellulose plus 50% (*w*/*w*) sugarcane bagasse. Letters A, B, C, D, E and F refer to points (Figure 9) where samples were taken for the EDS analysis.

Element	Mass Percentage (%)						
	A	B	C	D	E	F	Average Value
C	45.68	47.12	45.87	45.28	48.85	45.99	46.46 ± 1.32
O	48.17	47.41	47.78	47.90	44.09	47.42	47.13 ± 1.52
Zn	2.70	2.52	2.45	2.15	2.37	2.41	2.45 ± 0.18
Mg	-	-	-	0.14	0.27	0.24	0.22 ± 0.07
Al	-	-	-	0.21	0.55	-	0.38 ± 0.24
Si	-	-	0.53	0.47	0.70	0.18	0.47 ± 0.22
Cl	2.74	2.26	2.73	2.73	2.41	2.37	2.54 ± 0.22
K	-	-	-	-	-	0.12	0.12
Ca	0.70	0.70	0.63	1.12	0.76	1.26	0.86 ± 0.27

**Table 8:** Results of EDS analysis of the 0.7 BC/0.3 SCB composite composed of 70% (*w*/*w*) bacterial cellulose plus 30% (*w*/*w*) sugarcane bagasse. Letters A, B, C, D, E and F refer to points (Figure 10) where samples were taken for the EDS analysis.

Element	Mass Percentage (%)						
	A	B	C	D	E	F	Average Value
C	46.69	46.60	46.24	60.52	40.80	46.87	48.12 ± 6.57
O	46.63	47.42	46.63	33.81	46.15	46.80	44.57 ± 5.27
Zn	2.65	2.78	2.63	1.75	2.45	2.65	2.48 ± 0.37
Al	-	-	-	0.37	0.33	-	0.35 ± 0.03
Si	-	-	-	0.49	0.44	0.19	0.37 ± 0.16
Cl	2.59	2.57	2.96	1.88	2.53	2.45	2.49 ± 0.35
Ca	1.44	0.63	1.03	1.19	7.29	1.02	2.10 ± 2.52

**Table 9:** Samples and respective flexibility. BC = Bacterial cellulose, SCB = Sugarcane bagasse, BC/SCB = 50% (*w*/*w*) BC plus 50% (*w*/*w*) SCB, 0.7 BC/0.3 SCB = 70% (*w*/*w*) BC plus 30% (*w*/*w*) SCB.

Sample	Flexibility
BC	>100
SCB	26
BC/SCB	>100
0.7 BC/0.3 SCB	>100

## Data Availability

The original contributions presented in the study are included in the article, further inquiries can be directed to the corresponding authors.

## References

[1] Justin Koh J., Pang P., Chakraborty S., Kong J., Sng A., Anukunwithaya P., Huang S., Koh X.Q., Thenarianto C., Thitsartan W. (2023). Presence, origins and effect of stable surface hydration on regenerated cellulose for underwater oil-repellent membranes. J. Colloid Interface Sci..

[2] Hussain Z., Sajjad W., Khan T., Wahid F. (2019). Production of bacterial cellulose from industrial wastes: A review. Cellulose.

[3] Marestoni L.D., Barud H.d.S., Gomes R.J., Catarino R.P.F., Hata N.N.Y., Ressutte J.B., Spinosa W.A. (2020). Commercial and potential applications of bacterial cellulose in Brazil: Ten years review. Polímeros Ciência Tecnol..

[4] Muiruri J.K., Yeo JC C., Zhu Q., Ye E., Loh X.J., Li Z. (2023). Bacterial cellulose: Recent advances in biosynthesis, functionalization strategies and emerging applications. Eur. Polym. J..

[5] Teng C.P., Tan M.Y., Toh J.P.W., Lim Q.F., Wang X., Ponsford D., Lin E.M.J., Thitsartarn W., Tee S.Y. (2023). Advances in cellulose-based composites for energy applications. Materials.

[6] Singh A., Chandra R., Karina M., Souza T.C. (2019). Pollutants released from the pulp paper industry: Aquatic toxicity and their health hazards. Aquat. Toxicol..

[7] Andriani D., Apriyana A., Karina M., Souza T.C. (2020). The optimization of bacterial cellulose production and its applications: A review. Cellulose.

[8] Quintana E., Valls C., Roncero M.B. (2024). Valorization of waste-paper sludge as a sustainable source for packaging applications. Polym. Bull..

[9] Etuk S., Agbasi O., Robert U. (2021). Investigation of heat transfer and mechanical properties of *Saccharum officinarum* leaf board. Int. J. Energy Water Resour..

[10] Lekrine A., Belaadi A., Dembri I., Jawaid M., Ismail A.S., Abdullah M.M., Chai B.X., Al-khawlani A., Ghernaout D. (2024). Thermomechanical and structural analysis of green hybrid composites based on polylactic acid/biochar/treated *W. filifera* palm fibers. J. Mater. Res. Technol..

[11] Klemm D., Kramer F., Moritz S., Lindström T., Ankerfors M., Gray D., Dorris A. (2011). Nanocelluloses: A new family of nature-based materials. Angew. Chem. Int. Ed..

[12] Brown A.J. (1886). XIX.—The chemical action of pure cultivations of bacterium aceti. J. Chem. Soc. Trans..

[13] Carvalho T., Guedes G., Sousa F.L., Freire C.S.R., Santos H.A. (2019). Latest advances on bacterial cellulose-based materials for wound healing, delivery systems, and tissue engineering. Biotechnol. J..

[14] Gao H., Sun Q., Han Z., Li J., Liao B., Hu L., Huang J., Zou C., Jia C., Huang J. (2020). Comparison of bacterial nanocellulose produced by different strains under static and agitated culture conditions. Carbohydr. Polym..

[15] Amorim J.D.P., Nascimento H.A., Silva Junior C.J.G., Medeiros A.D.M., Silva I.D., Costa A.F.S., Vinhas G.M., Sarubbo L.A. (2021). Obtainment of bacterial cellulose with added propolis extract for cosmetic applications. Polym. Eng. Sci..

[16] Medeiros A.D.M., Silva Junior C.J.G., Amorim J.D.P., Durval I.J.B., Costa A.F.S., Sarubbo L.A. (2022). Oily wastewater treatment: Methods, challenges, and trends. Processes.

[17] Girard V.-d., Chaussé J., Vermette P. (2024). Bacterial cellulose: A comprehensive review. J. Appl. Polym. Sci..

[18] Płoska J., Garbowska M., Pluta A., Stasiak-Róŝańska L. (2023). Bacterial cellulose innovative biopolymer and possibilities of its applications in dairy industry. Int. Dairy J..

[19] Medeiros A.D.L.M., Silva Junior C.J.G., de Amorim J.D.P., do Nascimento H.A., Converti A., Costa A.F.S., Sarubbo L.A. (2021). Bacterial cellulose for treatment of wastewaters generated by energy consuming industries: A review. Energies.

[20] Vadanan S.V., Basu A., Lim S. (2022). Bacterial cellulose production, functionalization, and development of hybrid materials using synthetic biology. Polym. J..

[21] Singhania R.R., Patel A.K., Tseng Y.-S., Kumar V., Chen C.-W., Haldar D., Saini J.K., Dong C.-D. (2022). Developments in bioprocess for bacterial cellulose production. Bioresour. Technol..

[22] Kadier A., Ilyas R.A., Huzaifah M.R.M., Harihastuti N., Sapuan S.M., Harussani M.M., Azlin M.N.M., Yuliasni R., Ibrahim R., Atikah M.S.N. (2021). Use of Industrial Wastes as Sustainable Nutrient Sources for Bacterial Cellulose (BC) Production: Mechanism, Advances, and Future Perspectives. Polymers.

[23] Hegde S., Bhadri G., Narsapur K., Koppal S., Oswal P., Turmuri N., Jumnal V., Hungund B. (2013). Statistical optimization of medium components by response surface methodology for enhanced production of bacterial cellulose by *Gluconacetobacter persimmonis*. J. Bioprocess. Biotech..

[24] Domskiene J., Sederaviciute F., Simonaityte J. (2019). Kombucha bacterial cellulose for sustainable fashion. Int. J. Clothing Sci. Technol..

[25] Basu A., Vadanan S.V., Lim S. (2018). A novel platform for evaluating the environmental impacts on bacterial cellulose production. Sci. Rep..

[26] Ng F.M.C., Wang P.W. (2016). Natural self-grown fashion from bacterial cellulose: A paradigm shift design approach in fashion creation. Des. J..

[27] Rathinamoorthy R., Kiruba T. (2022). Bacterial cellulose-A potential material for sustainable eco-friendly fashion products. J. Nat. Fibers.

[28] Silva Junior C.J.G., Amorim J.D.P., Medeiros A.D.M., Cavalcanti A.K.L.H., Nascimento H.A., Henrique M.A., Maranhão L.J.C., Vinhas G.M., Souto Silva K.K.O., Costa A.F.S. (2022). Design of a naturally dyed and waterproof biotechnological leather from reconstituted cellulose. J. Funct. Biomater..

[29] Gupta H., Kumar H., Kumar M., Gehlaut A.K., Gaur A., Sacham S., Park J.-W. (2019). Synthesis of biodegradable films obtained from rice husk and sugarcane bagasse to be used as food packaging material. Environ. Eng. Res..

[30] Gond R.K., Gupta M.K. (2020). A novel approach for isolation of nanofibers from sugarcane bagasse and its characterization for packaging applications. Polym. Comp..

[31] Angelo J., Oliveira M., Ghobril C. (2021). Balança comercial dos agronegócios paulista e brasileiro de 2020. Análises Indicadores Agronegócio.

[32] Medeiros A.D.M.d., Silva Junior C.J.G.d., Amorim J.D.P.d., Durval I.J.B., Damian R.B., Cavalcanti Y.d.F., Costa A.F.d.S., Sarubbo L.A. (2023). Design and modeling of a biotechnological nanofiltration module using bacterial cellulose membranes for the separation of oily mixtures. Water.

[33] Kamiński K., Jarosz M., Grudzień J., Pawlik J., Zastawnik F., Pandyra P., Kołodziejczyk A.M. (2020). Hydrogel bacterial cellulose: A path to improved materials for new eco-friendly textiles. Cellulose.

[34] Chen G., Zhang B., Zhao J., Chen H. (2014). Development and characterization of food packaging film from cellulose sulfate. Food Hydrocol..

[35] Revin V.V., Liyas’kina E.V., Sapunova N.B., Bogatyreva A.O. (2020). Isolation and characterization of the strains producing bacterial cellulose. Microbiology.

[36] Costa A.F.S., Almeida F.C.G., Vinhas G.M., Sarubbo L.A. (2017). Production of bacterial cellulose by *Gluconacetobacter hansenii* using corn steep liquor as nutrient sources. Front. Microbiol..

[37] Nascimento H.A., Amorim J.D.P., Silva C.J.G.J., Medeiros A.D.M., Costa A.F.S., Napoleão D.C., Vinhas G.M., Sarubbo L.A. (2022). Influence of gamma irradiation on the properties of bacterial cellulose produced with concord grape and red cabbage extracts. Curr. Res. Biotechnol..

[38] Zhou L., Fu J., Bian L., Chang T., Zhang C. (2022). Preparation of a novel curdlan/bacterial cellulose/cinnamon essential oil blending film for food packaging application. Int. J. Biol. Macromol..

[39] Yoshimitsu Z., Nakajima A., Watanabe T., Hashimoto K. (2002). Effects of surface structure on the hydrophobicity and sliding behavior of water droplets. Langmuir.

[40] Xiong J., Sheng C., Wang Q., Guo W. (2019). Toughened and water-resistant starch/TiO_2_ bio-nanocomposites as an environment-friendly food packaging material. Mater. Res. Exp..

[41] Ul-Islam M., Khan S., Ullah M.W., Park J.K. (2019). Comparative study of plant and bacterial cellulose pellicles regenerated from dissolved states. Int. J. Biol. Macromol..

[42] Halder P., Kundu S., Patel S., Parthasarathy R., Pramanik B., Paz-Ferreiro J., Shah K. (2019). TGA-FTIR study on the slow pyrolysis of lignin and cellulose-rich fractions derived from imidazolium-based ionic liquid pre-treatment of sugarcane straw. Energy Convers. Manag..

[43] Frone A.N., Panaitescu D.M., Chiulan I., Nicolae C.A., Casarica A., Gabor A.R., Trusca R., Damian C.M., Purcar V., Alexandrescu E. (2018). Surface treatment of bacterial cellulose in mild, eco-friendly conditions. Coatings.

[44] Zhou H., Long Y., Meng A., Chen S., Li Q., Zhang Y. (2015). A novel method for kinetics analysis of pyrolysis of hemicellulose, cellulose, and lignin in TGA and macro-TGA. RSC Adv..

[45] Tsouko E., Kourmentza C., Ladakis D., Kopsahelis N., Mandala I., Papanikolaou S., Paloukis F., Alves V., Koutinas A. (2015). Bacterial cellulose production from industrial waste and by-product streams. Int. J. Mol. Sci..

[46] Hamed D.A., Maghrawy H.H., Kareem H.A. (2023). Biosynthesis of bacterial cellulose nanofibrils in black tea media by a symbiotic culture of bacteria and yeast isolated from commercial kombucha beverage. World J. Microbiol. Biotechnol..

[47] Digel I., Akimbekov N., Rogachev E., Pogorelova N. (2023). Bacterial cellulose produced by *Medusomyces gisevii* on glucose and sucrose: Biosynthesis and structural properties. Cellulose.

[48] Liu X., Xu Y., Liao W., Guo C., Gan M., Wang Q. (2023). Preparation and characterization of chitosan/bacterial cellulose composite biodegradable films combined with curcumin and its application on preservation of strawberries. Food Packag. Shelf Life.

[49] Ul-Islam M., Alhajaim W., Fatima A., Yasir S., Kamal T., Abbas Y., Khan S., Khan A.H., Manan S., Ullah M.W. (2023). Development of low-cost bacterial cellulose-pomegranate peel extract-based antibacterial composite for potential biomedical applications. Int. J. Biol. Macromol..

[50] Mohammadkazemi F., Doosthoseini K., Azin M. (2014). Effect of ethanol and medium on bacterial cellulose (BC) production by *Gluconacetobacter xylinus* (PTCC 1734). Cellul. Chem. Technol..

[51] Fatima A., Ortiz-Albo P., Neves L.A., Nascimento F.X., Crespo J.G. (2023). Biosynthesis and characterization of bacterial cellulose membranes presenting relevant characteristics for air/gas filtration. J. Membr. Sci..

[52] Grande C.J., Torres F.G., Gomez C.M., Bañó M.C. (2009). Nanocomposites of bacterial cellulose/hydroxyapatite for biomedical applications. Acta Biomater..

[53] Kapoor K., Tyagi A.K., Das M., Kumar V. (2023). Comparative morphological and structural changes in gamma and electron beam irradiated sugarcane bagasse. Cellulose Chem. Technol..

[54] Maragongi Junior L., Cristianini M., Padula M., Anjos C.A.R. (2019). Effect of high-pressure processing on characteristics of flexible packaging for foods and beverages. Food Res. Int..

[55] Wu X., Mou H., Fan H., Yin J., Liu Y., Liu J. (2022). Improving the flexibility and durability of aged paper with bacterial cellulose. Mater. Today Commun..

[56] Afrifah K.A., Adom A.N.A., Ofosu S. (2022). The morphological and pulping indices of bagasse, elephant grass (leaves and stalk), and silk cotton fibers for paper production. J. Nat. Fibers.

